# DYRK1A in blood and immune function: implications in leukemia, inflammatory disorders, infection and Down syndrome

**DOI:** 10.3389/fcell.2025.1587089

**Published:** 2025-05-30

**Authors:** Esteban J. Rozen, Robin D. Dowell, Mary A. Allen

**Affiliations:** ^1^ Crnic Institute Boulder Branch, BioFrontiers Institute, University of Colorado Boulder, Boulder, CO, United States; ^2^ Department of Molecular, Cellular and Developmental Biology, University of Colorado Boulder, Boulder, CO, United States

**Keywords:** trisomy 21, Down syndrome, DYRK1A, blood and immune function, leukemia, inflammation, viral infection

## Abstract

Down syndrome (DS) is the most frequent autosomal aneuploidy, and it arises due to an extra copy of human chromosome 21. Individuals with trisomy 21 (T21) exhibit an increased predisposition towards a wide number of developmental and physiological alterations, often referred to as DS co-occurring conditions, including congenital heart disease, leukemia, intellectual disability, neurodegenerative disorders or autoimmune diseases, among many others. The overexpression of several genes encoded on chromosome 21 have been linked to many of such T21-associated disorders, but we are still very far from grasping a full picture of the contributions and interconnections of such genes in the pathophysiology of DS. DYRK1A is a versatile and ubiquitous kinase encoded on human chromosome 21, and as such, its activity has been linked to many alterations that characterize DS. Although most of the attention has been focused on DYRK1A’s roles in neural development, function and degeneration, accumulating reports are expanding the scope towards other tissues and conditions where this kinase also performs critical functions, such as the cardiovascular system, diabetes, inflammation and immune homeostasis. Here, we present a detailed review of the literature summarizing all the information linking DYRK1A to blood and immune function, as well as leukemia, inflammation and viral infections, with a special focus on their potential associations to T21. This article synthesizes evidence that supports several novel hypotheses on previously unsuspected roles for DYRK1A in specific DS alterations, opening new pathways for the research community to explore and therefore, contributing to future innovative diagnostic or therapeutic interventions. This article will hopefully inspire and guide the advancement of our knowledge leading to much needed treatments for individuals with Down syndrome, but also for the general population.

## Introduction

Triplication of human chromosome 21 (Hsa21) –or Trisomy 21– is the genetic cause of Down syndrome (DS), the most frequent autosomal aneuploidy in humans. With a prevalence of ∼1 in 700 births (Lagan et al., 2020; Antonarakis et al., 2020), Trisomy 21 (T21) results in a wide array of developmental and pathophysiological alterations, including intellectual disability, craniofacial and musculoskeletal alterations, blood and immune dysregulation, early-onset alzheimer’s disease, and congenital heart defects (CHDs) (Lagan et al., 2020; Antonarakis et al., 2020). It is hypothesized–and in some cases confirmed–that an extra copy of one or several genes encoded on Hsa21 predisposes to–or directly promotes–specific co-occurring conditions in individuals with DS, although in many instances the identities and implications of such genes remain poorly characterized (Scarpato et al., 2014; Antonarakis, 2017). The identification of disease-causing genes has undoubtedly enabled a better understanding of the underlying pathological mechanisms, resulting in improved strategies for diagnosis, management and treatment of DS-associated disorders, for which more effective and personalized therapeutic alternatives are still urgently needed.

Individuals with DS have a high incidence of blood and immune alterations, including autoimmune disorders and leukemias–particularly Acute Megakaryoblastic Leukemia (AMKL) and B-cell Acute Lymphoid Leukemia (B-ALL)–. Several protein-coding genes on Hsa21 have been shown to predispose for these disorders, among which a cluster of four interferon (IFN) receptor genes (IFNAR1, IFNAR2, IFNGR2 and IL10RB), the hematopoietic transcription factor RUNX1 and the DYRK1A kinase appear to play critical roles. DYRK1A (Dual-specificity and tYrosine phosphorylation-regulated Kinase 1A) has emerged as a pleiotropic regulator of various cellular processes, including neural development and activity, neurodegeneration, immunity, and cardiac development and function, among several other roles (Ananthapadmanabhan et al., 2023; Yang et al., 2023; Deboever et al., 2022). Noteworthy, an accumulating body of literature delineates a plethora of functions for this kinase in blood and immune cell homeostasis, as well as in leukemia, inflammation and viral infections, all of which may have direct implications into our understanding of DS co-occurring disorders. However, a comprehensive review of these data is lacking. Here, we provide a succinct synthesis of the current knowledge on the contributions of DRK1A to blood and immune function, leukemia, inflammation and viral responses, aiming at facilitating thoughtful interpretations by the research community and inspiring better informed future efforts in these fields.

### Blood, inflammation and leukemia in trisomy 21

Children with DS exhibit alterations in blood cell development and function, whose consequences in dysregulated immune responses and inflammation are very well documented. Thus, excellent reviews on this topic can be found elsewhere (Roberts and Izraeli, 2014; Araya et al., 2019; Waugh et al., 2019; Jardine et al., 2021; Dieudonné et al., 2020; Ramba and Bogunovic, 2024; Lambert et al., 2022). For that reason, we will only introduce some general conclusions from the abundant literature. The full blood and immune cell repertoire is established in the fetal bone marrow (FBM) in a short time window of 6–7 weeks early during the second trimester of embryonic development. In this specific microenvironment myeloid progenitors undergo efficient proliferation and differentiation into diverse fates, such as granulocytes, eosinophils and dendritic cells. T21 progenitor cells exhibit specific impairments in B-lymphocyte, erythroid and myeloid development, likely due to a cell-intrinsic bias favoring the erythroid lineage at the expense of myeloid and B-cell colonies, and possibly due to a more pro-inflammatory microenvironment (Jardine et al., 2021). Postnatally, individuals with DS exhibit alterations on virtually every cell subpopulation of the innate and adaptive immune systems, as well as increased levels of pro-inflammatory cytokines, a shift toward memory-like T-cells, and a reduced B-cell compartment, which predisposes people with DS to more frequent autoimmunity and more severe infections (Ramba and Bogunovic, 2024). Several reports have shown that adults with DS display a global immune dysregulation, including key changes in the myeloid and lymphoid cell compartments, consistent with hypersensitive IFN signaling and chronic inflammation (Araya et al., 2019; Waugh et al., 2019; Tuttle et al., 2020). In parallel, Malle and others (Malle et al., 2023) confirmed chronic IL-6 signaling in CD4^+^ T-cells from individuals with T21 –as previously reported by others (Lambert et al., 2022; Sullivan et al., 2017)–, as well as higher proportions of CD11c^+^ B-cell plasmablasts. The latter subpopulation may be associated with autoimmune reactions, further supported by the identification of 365 unique auto-antibodies in the plasma of individuals with DS, delineating a novel IFN-independent mechanism underlying autoimmune predisposition in DS.

Additionally, newborns with T21 display a much higher incidence of acute megakaryoblastic leukemia (AMKL; up to 500-fold) and acute lymphoblastic leukemia (ALL; 20–30-fold) compared to euploid children. This has been the focus of intense research for many years, and thus, excellent reviews on this topic are available (Roberts and Izraeli, 2014; Malinge et al., 2009; Schmidt et al., 2021; Gupte et al., 2022; Baruchel et al., 2023; Mason et al., 2024). Therefore, here we will offer only a brief summary of the general aspects of DS-associated leukemia. Epidemiological studies show that about 10% of children with DS are born with transient myeloproliferative disorder (TMD), a clonal pre-leukemic condition characterized by an accumulation of immature megakaryoblasts in the fetal liver and peripheral blood (Schmidt et al., 2021). While in most children with T21 TMD resolves spontaneously, in 20%–30% of these patients some clones reemerge as AMKL within ∼4 years (Schmidt et al., 2021; Mateos et al., 2015). Of note, virtually all individuals with DS who are born with TMD and/or who develop AMKL exhibit truncating mutations of the transcription factor *GATA1* gene–collectively referred to as *GATA1s*–. Surprisingly, GATA1s mutations confer increased risk of leukemia only in the context of T21, but not in euploid individuals.

Altogether, our understanding of blood and immune system development, function and dysregulations in people with DS is growing at an unprecedented rate, leading to novel translational innovations that are poised to transform the treatment of immune disorders in this population and beyond. However, we are still far from getting a grasp on the full picture of the intertwined mechanisms underlying these phenotypes, their regulation and complexity. As we will describe in the following sections, the Hsa21-encoded DYRK1A kinase is emerging as central player in the modulation of blood and immune cells homeostasis, as well as in the pathways that drive inflammation, leukemia and viral infections.

### DYRK1A and trisomy 21

DYRK1A is a ubiquitous and pleiotropic enzyme, modulating the activity of hundreds of proteins, signaling pathways and transcriptional events. We refer the reader to several excellent and comprehensive reviews that summarize our understanding on DYRK1A’s molecular and cellular functions, and its implications in health and disease (Ananthapadmanabhan et al., 2023; Yang et al., 2023; Deboever et al., 2022; Rammohan et al., 2022), as well as its specific roles in DS (Laham et al., 2021; Kay et al., 2016; Stringer et al., 2017; Atas-Ozcan et al., 2021; Murphy et al., 2024). In summary, DYRK1A is a member of the DYRK family of protein kinases, which belongs to the CMGC kinase superfamily (Cyclin-dependent kinases, Mitogen-activated protein kinases, Glycogen synthase kinases, CDC-like kinases). It is characterized by its dual-specificity, autophosphorylating on tyrosine residues during maturation and phosphorylating serine/threonine residues on its substrates. DYRK1A’s subcellular distribution can vary depending on expression levels, cell type and cell cycle/developmental stage. In general, it is predominantly localized in the cytoplasm, but it can shuttle the nucleus upon over-expression. Functionally, DYRK1A is involved in a wide array of cellular processes including neurodevelopment, cell proliferation, and apoptosis. It phosphorylates multiple substrates such as cyclin D1, NFAT (nuclear factor of activated T-cells), or RNA polymerase II, affecting their stability, localization, or activity. Through these actions, DYRK1A plays a pivotal role in transcriptional and signaling regulation including those related to brain development and synaptic function (reviewed in (Yang et al., 2023; Deboever et al., 2022)). It is encoded in 21q22.2, within the so-called “Down syndrome Critical Region” (DSCR) of human Chromosome 21 (Pelleri et al., 2016; Pelleri et al., 2019), triplication of which is necessary and sufficient for the manifestation of multi-organ developmental abnormalities, facial gestalt, intellectual disability, and early-onset alzheimer’s disease in individuals with DS (Martinez De Lagran et al., 2012; Park and Chung, 2013). Initial studies demonstrated a crucial role for DYRK1A in neural development and function, conserved through evolution from flies to humans (Kay et al., 2016; Fischbach and Heisenberg, 1984). Hence, the direct association between DYRK1A dosage imbalance and intellectual disability in T21 has been the focus of intense investigation for 40 years (Ananthapadmanabhan et al., 2023; Yang et al., 2023; Deboever et al., 2022; Rammohan et al., 2022; Laham et al., 2021; Kay et al., 2016; Stringer et al., 2017; Atas-Ozcan et al., 2021; Murphy et al., 2024). Here, we present a detailed review of the literature reporting on specific roles of DYRK1A in blood and immune cell development and function, as well as in leukemia, inflammatory disorders, and viral infections. The implications or possible associations of these mechanisms to T21 pathophysiology are also discussed. This compendium of information will undoubtedly lead to new hypotheses and improved interpretations in the field, allowing novel innovations for the management and treatment of Down syndrome co-occurring conditions.

### DYRK1A in blood development and immune responses

Accumulating work from many groups highlights diverse roles for DYRK1A as a critical regulator of several pathways related to blood and immune cell function, particularly in the context of lymphocytes and the adaptive immune system. Initial evidence suggesting a role for DYRK1A in blood cells was provided by Dowjat et al. (Dowjat et al., 2012), who assessed the interaction of DYRK1A with cytoskeletal proteins in immortalized B lymphocytes (lymphoblastoid cells) isolated from individuals with or without DS. The study found that specific DYRK1A overexpression led to reduced interaction with β-actin, indicating that DYRK1A dosage might regulate lymphocyte mechanics and function in T21. In a follow-up study, the authors confirmed these observations and suggested that such reduced interaction between DYRK1A and cytoskeletal proteins may constitute an early biomarker for the diagnosis of Alzheimer’s disease from PMBCs (Dowjat et al., 2019).

#### DYRK1A in T-cell differentiation and function

In 2015, two groups reported more detailed insights into the functions of DYRK1A in lymphocytes. On one hand, Thompson et al. (2015) demonstrated a crucial role for this kinase in the regulation of Cyclin D3 stability, which is necessary for the differentiation of pre-B and pre-T lymphocyte precursors. Specifically, they showed that DYRK1A induces Cyclin D3 degradation by phosphorylating it on Threonine-283. Genetic or pharmacologic inhibition DYRK1A activity in mice led to Cyclin D3 accumulation, sustained activity of E2F-dependent transcription–a master regulator of cell cycle genes–, and failure to exit the cell cycle and commit to differentiation, hence resulting in reduced numbers of mature B-cells–while not affecting myeloid development. On the other hand, Khor et al. (2015) observed that inhibition of DYRK1A enhanced the differentiation of anti-inflammatory regulatory CD4^+^ T (T_reg_) cells, while impairing differentiation of pro-inflammatory T_h17_ helper cells through a novel mechanistic node at the branch point between commitment to either lineage, independently of the canonical signaling pathways that drive each of these populations. Importantly, the DYRK1A inhibitor harmine potently attenuated inflammation in multiple experimental murine models of systemic autoimmunity and mucosal inflammation. Nevertheless, caution should be taken when interpreting these results, as harmine has several off-target effects. Work from Valencic et al. (2019) partially challenged this idea by analyzing the immune compartments of two patients with mental retardation disorder 7 (MRD7) –an autosomal dominant intellectual disability caused by DYRK1A haploinsufficiency–vs. one healthy control. The authors did not find significant differences in the number of T_reg_ and T_h17_ populations, although important experimental caveats–e.g., samples derived from only two patients of widely disparate ages, or incomplete disruption of DYRK1A expression–limit the interpretation of these results. Importantly, these studies did not address the potential roles of DYRK1A overexpression–as is the case in T21-derived lymphocytes–, although Khor et al. (2015) did speculate on the fact that individuals with DS exhibit hypofunctional T_reg_ cells and show an increased incidence of autoimmune conditions (Araya et al., 2019; Malle et al., 2023; Pellegrini et al., 2012).

DYRK1A is known to phosphorylate and suppress the nuclear localization and transcriptional activity of the nuclear factor of activated T-cells (NFAT) family of transcription factors (Arron et al., 2006). Along these lines, Giri et al. (2023) reported that harmine–in combination with the FOXP3 activator Kaempferol–enhanced NFATC1/FOXP3-mediated T_reg_’s suppressive capacity in vitiligo models, leading to reduced CD8^+^ and CD4^+^ T-cell proliferation, reduced IFN-γ production, and increased melanocyte survival and proliferation. In a previous study, this group had confirmed that NFATc1 signaling is crucial for T_reg_ differentiation in this model and observed that increased *DYRK1A* and *GSK3B* transcripts lead to decreased NFATc1 activity, which is responsible for reduced T_reg_ suppressive capacity (Giri et al., 2022), thus highlighting the role of DYRK1A in autoimmune disease severity and progression. Accordingly, Kim et al. (2023) identified a novel, potent and selective inhibitor of DYRK1A, FRTX-02, which induced transcriptional activity of NFAT in T-cell lines. Correspondingly, FRTX-02 promoted *ex vivo* CD4^+^ polarization into anti-inflammatory T_reg_ cells and reduced their fate towards pro-inflammatory T_h1_ or T_h17_ cells. Finally, in mouse models of psoriasis and atopic dermatitis, FRTX-02 reduced inflammation and disease biomarkers in a dose-dependent manner–leading to a Phase 1 clinical trial (NCT05382819)–, and confirming the utility of DYRK1A inhibitors, such as FRTX-02, as potential therapies for chronic inflammatory and autoimmune conditions. More recently, Malueg et al. (2025) confirmed that DYRK1A regulates T_h17_ differentiation at least in part by tunning the responsiveness to IL-6 by directly regulating the surface expression of IL-6 receptor subunits gp130 and IL-6R. Of note, gp130 acts as a co-receptor for several other cytokines from the IL-6 family (Rose-John, 2018), underlying many other potential functions. In this line, the authors found that DYRK1A-regulated expression of IL-6 receptor subunits extends beyond naïve CD4^+^ T-cell subsets. Consistent with these findings, Qi et al. (2023) reported that miR-1246 – previously shown to target *DYRK1A*’s 3′-UTR–was significantly decreased in peripheral CD4^+^ T-cells of patients with severe active Alopecia Areata. Accordingly, ectopic expression of DYRK1A in these cells resulted in higher proportion and function of T_h17_ cells, while overexpression of miR-1246 had the opposite effects. Although extreme caution should be taken when interpreting experiments using small molecules–subject to potential off-target effects–, all these data agree with reports suggesting decreased T_reg_ function and increased risk of autoimmunity in people with DS (Araya et al., 2019; Malle et al., 2023; Pellegrini et al., 2012), and allude to the plausible use of DYRK1A inhibitors as an innovative path for the treatment of T21-associated auto-inflammatory conditions. Importantly, most of the experiments linking DYRK1A to T_reg_ differentiation defects are based on small-molecule inhibitors–and therefore subject to potential off-target effects. Future work must provide genetic evidence to confirm DYRK1A regulation of T_reg_ fate.

#### DYRK1A in B-cell differentiation and function

Stoler-Barak et al. (2023) recently reported that DYRK1A is essential for B-cell-mediated protection from viral infection and effective vaccination through regulation of class switch recombination (CSR), by a mechanism involving direct phosphorylation of the DNA mismatch repair protein MSH6. Importantly, the study also found that DYRK1A is required for attenuation of B-cell proliferation in germinal centers at later stages of the response through negative regulation of multiple cell-cycle factors. Over-expression experiments were not performed in this context, only allowing for the speculation that increased levels of DYRK1A in germinal center B-cells might induce premature cell cycle exit and reduced clonal expansion, consistent with previous reports showing lower numbers of certain B-cell subpopulations in people with T21 (Waugh et al., 2019; Jardine et al., 2021; Ramba and Bogunovic, 2024; Farroni et al., 2018; Verstegen et al., 2014).

B-cell-activating factor (BAFF) is a cytokine that mediates B-cell survival and, when dysregulated, contributes to autoimmune diseases and B-cell malignancies. Li et al. (2021) identified DYRK1A as a kinase that responds to BAFF stimulation and mediates BAFF-induced B-cell survival, while DYRK1A deficiency in these cells caused peripheral B-cell reduction and ameliorated autoimmunity in a mouse model of lupus. Mechanistically, they showed that DYRK1A phosphorylates TRAF3 at Serine-29 to interfere with its function in mediating degradation of the noncanonical NF-κB-inducing kinase (NIK), thereby facilitating BAFF-induced NIK accumulation and alternative NF-κB activation. Finally, the authors linked this novel DYRK1A function to B-cell acute lymphoblastic leukemia pathogenesis (see next section).

#### DYRK1A in other blood cell types

Beyond lymphocytes, Elagib et al. (2022) reported that DYRK1A inhibition induced enlargement, polyploidization, and thrombopoiesis of human neonatal and induced pluripotent stem cell (iPSC)-derived megakaryocytes, through a mechanism involving direct regulation of the actin-regulated transcriptional co-activator MKL1, hence identifying DYRK1A as a critical negative regulator of megakaryocyte differentiation with important clinical implications. As described in the next section, these observations agree with a previous report supporting a prominent role for DYRK1A as a potent megakaryoblastic tumor-promoting gene that contributes to DS-associated AMKL leukemogenesis (Malinge et al., 2012). Furthermore, Postic et al., (2023) investigated platelet function and bleeding in a transgenic murine strain overexpressing Dyrk1A (mBACtgDyrk1A). Mice with Dyrk1A overexpression had a ∼20% reduction in platelet numbers, while bleeding time was decreased by around 50%. These phenotypes were not associated with abnormal expression of platelet receptors, nor to defects on platelet activation or half-life. Finally, Dyrk1A-overexpressing mice showed increased levels of plasma fibronectin and fibrinogen, which was associated to enhanced production hepatic fibrinogen.

Using zebrafish overexpressing the human *DYRK1A* gene, Liu Y. et al. (2022) observed significant alterations during early development of the embryonic organizer and body axis, resulting in defects in blood and several other tissues, reminiscent of those observed in individuals with DS. Phosphoproteomic analysis showed that DYRK1A-overexpressing fish embryos had anomalous phosphorylation of β-catenin and Hsp90ab1, resulting in enhanced Wnt signaling and inhibition of TGF-β. This pattern was confirmed in blood hematopoietic stem cells (HSCs) from individuals with DS. Importantly, the abnormal proliferation of DS-derived HSCs could be recovered by switching the balance between Wnt and TGF-β signaling *in vitro*.

Waugh et al. (2023) correlated the overexpression of Hsa21 genes and immune markers across the lifespan using matched whole-blood transcriptome and plasma immune marker data from 304 individuals with T21 versus 96 euploid controls. Despite the evidence supporting distinct roles for DYRK1A in immune and inflammatory pathologies, the authors did not observe positive correlations between DYRK1A and multiple inflammatory pathways and immune markers (such as CSTB, C-reactive protein or IL-6), suggesting a minor role for DYRK1A in chronic interferon hyperactivity and inflammation in people with DS.

**FIGURE 1:**
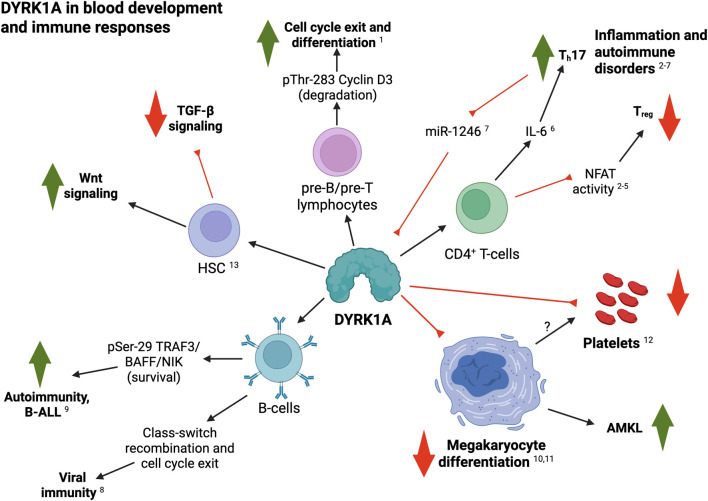
Summary of DYRK1A roles in blood and immune cell development and function. Black arrows indicate a positive regulatory role; red lines indicates inhibitory interactions (see text for details; created with BioRender). 1, Thompson et al. (2015); 2, Khor et al. (2015); 3, Giri et al. (2022); 4, Giri et al. (2023); 5, Kim et al. (2023); 6, Malueg et al. (2025); 7, Qi et al. (2023); 8, Stoler-Barak et al. (2023); 9, Li et al. (2021); 10, Elagib et al. (2022); 11, Malinge et al. (2012); 12, Postic et al. (2023); 13, Liu et al. (2022a).

In summary, accumulating data supports pleiotropic functions for DYRK1A in the regulation of blood and immune cell homeostasis, differentiation and function (Figure 1). This is particularly relevant for B- and T-cells, where dysregulation of DYRK1A–i.e., due to T21– may underly important pathogenic outcomes associated with DS, such as uncontrolled (auto-) inflammatory conditions or leukemia (see next sections). Similarly, these mechanisms have opened the door to the development of promising therapeutic interventions targeting DYRK1A function or expression, some of which are currently being tested in clinical trials. While much attention has been focused on the implications of exacerbated IFN signaling in DS-related innate immune disorders, we posit that these data should be revisited in the context of potential synergies with DYRK1A-dependent mechanisms driving or potentiating adaptive immune alterations.

### DYRK1A in leukemia

Children with T21 show up to 500-fold higher risk of developing acute megakaryoblastic leukemia (AMKL) and ∼20-fold higher incidence of B-cell acute lymphoblastic leukemia (B-ALL) (Malinge et al., 2009; Schmidt et al., 2021; Mason et al., 2024). About 10% of children with DS are born with pre-leukemic transient myeloproliferative disorder (TMD) (Schmidt et al., 2021). Of these, about 20%–30% progress into AMKL (Schmidt et al., 2021; Mateos et al., 2015). Truncating mutations on GATA1 –so-called GATA1s mutants–are necessary, but not sufficient, for the manifestation of TMD and AMKL in individuals with DS.

#### DYRK1A in DS-AMKL

To elucidate the impact of T21 in leukemogenesis, Malinge and colleagues (Malinge et al., 2012) used human cells and mouse models of DS to reproduce the multistep pathogenesis of DS-AMKL, while uncovering Hsa21 genes that predispose to DS-AMKL. Using the Ts1Rhr mouse model of DS revealed that trisomy of just 33 Hsa21 ortholog genes–including Dyrk1A–was sufficient to cooperate with GATA1s mutations and initiate AMKL *in vivo*. They further observed that pediatric samples of DS-TMD and DS-AMKL expressed significantly higher levels of DYRK1A when compared to AML and AMKL samples without T21. Cell-based experiments demonstrated that DYRK1A was a potent megakaryoblastic tumor-promoting gene that contributes to leukemogenesis through dysregulation of NFAT signaling. These observations agree with a case report of a female infant with partial T21 presenting TMD (Takahashi et al., 2015). SNP array analysis showed amplification of a 10 Mb region between 21q22.12–21q22.3, including DYRK1A–among several other genes–but excluding RUNX1, another Hsa21 gene implicated in DS-AMKL (Gialesaki et al., 2023; Rozen et al., 2023).

Jang et al. (2014) reported that DYRK1A-dependent phosphorylation of Threonine-45 and Serine-57 on histone H3 differentially affects binding of the three mammalian heterochromatin protein 1 (HP1) paralogs HP1α, HP1β and HP1γ. H3 phosphorylation by DYRK1A impaired HP1-mediated transcriptional repression of pro-inflammatory genes in cellular models and in DS-associated megakaryoblastic leukemic cells. Surprisingly, a recent study by Sit et al. (2023) found that DYRK1A genetic ablation in a T21 human iPSC line harboring the GATA1s mutation resulted in increased megakaryocyte proliferation and decreased maturation, while wild-type (wt) GATA1-expressing isogenic T21 cells did not show significant changes. Nevertheless, day-7 CD43^+^ progenitors generated from both T21/GATA1wt and T21/GATA1s lines did show a decrease in absolute numbers when DYRK1A was disrupted (vs. DYRK1A trisomic counterparts). Based on these findings, the authors proposed that–contrary to previous observations in human and mouse models of T21–, DYRK1A (in synergy with GATA1s) may restrain megakaryocyte proliferation in human T21 cells. This suggests potential species-specific differences between human and mouse, although the lack of important controls (such as euploid isogenic controls) and the nature of the experimental models and approaches used in each case do not allow broader interpretations. Moreover, these observations were recently challenged by Chen et al. (2024), who found that individuals with T21 show increased chromosomal copy number variations (CNVs) compared to euploid individuals, leading to the hypothesis that genome instability could underly the predisposition to TAM and AML in this population. They then established a TAM disease model utilizing T21 and isogenic euploid human iPSCs, with which they generated GATA1s-mutant lines by genome editing. Using this system, they observed that acquisition of GATA1s enforces myeloid skewing and maintenance of the hematopoietic progenitor state independently of T21. More importantly, GATA1s in T21 hematopoietic progenitor cells (HPCs) further augmented genome instability. Finally, the authors demonstrated that increased dosage of DYRK1A impairs homology-directed DNA repair as a driving mechanism of elevated mutagenesis and transformation.

Contrary to pediatric DS-AMKL, in acute myeloid leukemia (AML) cells from euploid adults, DYRK1A could exert a tumor suppressor function. In this context, Liu et al. (2014) noted that DYRK1A expression level was reduced in the bone marrow of adult AML patients, compared to healthy controls. Overexpression of DYRK1A inhibited the proliferation of AML cell lines by increasing the proportion of cells in G0/G1 phase. The authors proposed that reduced proliferation by DYRK1A was mediated by induction of c-Myc degradation. Thus, overexpression of c-Myc markedly reversed AML cell growth inhibition induced by DYRK1A.

#### DS-ALL and DYRK1A

B-ALL is the most frequent pediatric cancer, typically diagnosed in children between 2 and 5 years-old (Ward et al., 2014). Groundbreaking innovations in the treatment of B-ALL have resulted in overall 5-year event-free survival (EFS) rates higher than 85% (Hunger et al., 2013). As mentioned previously, children with DS have a 20-30x higher risk of developing ALL (DS-ALL) (Hasle et al., 2000), almost exclusively of B-cell origin, while displaying mildly–but significantly–lower survival rates (Buitenkamp et al., 2014). In addition to trisomy of Hsa21, DS-ALL blasts can present heterogeneous genetic alterations, including JAK2-activating mutations, CRLF2 overexpression, and loss of IKZF1 and PAX5 (Lee et al., 2016). In this context, Bhansali et al. (2021) reported that DYRK1A is necessary for the growth of B-ALL cells, through mechanisms involving direct phosphorylation of FOXO1 –modulating DNA damage responses–and STAT3 –likely through the regulation of Reactive Oxygen Species production–. Lastly, they demonstrated that DYRK1A, FOXO1, and STAT3 can be effectively targeted by selective and potent small-molecule inhibitors as a novel therapeutic avenue for B-ALL in children with and without T21.

In a follow-up study, Carey-Smith et al., (2022) established two murine DS-ALL cell lines (Tc1-KRASG12D and Tc1-BCR-ABL) and disomic controls (WT-KRASG12D and WT-BCR-ABL). DS cells were derived from the Tc1 transchromosomic mouse model, harboring a freely segregating fragment of human chromosome 21 containing approximately 90% of Hsa21 genes. shRNA-mediated downregulation of Dyrk1a in these cells resulted in significant reduction of cell numbers, with trisomic cells showing increased sensitivity compared to euploid controls. They next analyzed the effect of different DYRK1A inhibitors–namely EHT1610 (Chaikuad et al., 2016), Leucettinib-21 (Deau et al., 2023), AM30 and AM45 (Zhou et al., 2017)– in *in vitro* cell proliferation assays. Leucettinib-21, AM30 and AM45 were more potent than EHT1610 at decreasing cellular growth in all cell lines tested, with Tc1-KRASG12D cells again displaying increased sensitivity. Finally, the authors established two human DS-ALL cell lines, DS-PER961 and DS-PER962, from previously reported PDX models (Laurent et al., 2020). Using these human cells, they confirmed that Leucettinib-21, AM30 and AM45 were potent inhibitors of viability in human DS-ALL cells and in MHH-CALL4 (a non-DS ALL cell line). Leucettinib-21 was the most potent compound to inhibit DYRK1A-mediated Cyclin D3 phosphorylation in a dose-dependent manner. Lastly, they assessed the efficacy of Leucettinib-21 in two DS-ALL PDX mouse models and observed that the treatment significantly decreased leukemic burden *in vivo*.

#### Non-DS-ALL and DYRK1A

A recent study by Jin et al. (2024) reported that in relapsed/refractory B-ALL models, pharmacologic blockade of DYRK1A–and potentially other CMGC kinases–through EHT1610 and GNF2133 (Liu et al., 2020), led to substantial changes in the alternative splicing of splicing factor transcripts such as *RBM39*. Specifically, DYRK1A inhibition resulted in the inclusion of a “poison” exon in the nascent *RBM39* mRNA, which is recognized by the nonsense-mediated mRNA decay pathway for degradation, resulting in RBM39 protein downregulation and subsequent cell death.

As stated earlier, Li et al. (2021) showed that DYRK1A mediates B-cell-activating factor (BAFF)-induced survival of normal B-cells, while DYRK1A deficiency lowered peripheral B-cell numbers and reduced autoimmune burden in a murine lupus paradigm. In detail, DYRK1A suppressed NIK degradation by phosphorylating TRAF3 Ser-29, thereby promoting BAFF-dependent NIK accumulation and alternative NF-κB activation. Importantly, the authors confirmed the role of this novel DYRK1A/NIK signaling cascade in B-cell ALL cell survival and *in vivo* pathogenesis, while demonstrating the potential of DYRK1A inhibitors, such as EHT1610, for B-cell ALL therapy.

KMT2A-Rearragend B-ALL is a high-risk genomic subtype that affects more than 70% of new B-ALL diagnoses in infants (<1 year of age), 5%–6% of pediatric cases and 15% of adult cases (Górecki et al., 2023), and originally referred to as ‘mixed lineage leukemia’. Ayyadevara et al. (2025), Ayyadevara et al., (2022) performed a kinome-wide CRISPR screen and identified DYRK1A as a factor required for KMT2A-R ALL cell survival, but not in other high-risk ALL subtypes. Pharmacologic inhibition of DYRK1A with the specific inhibitory compound EHT1610 demonstrated potent inhibition of leukemic cell proliferation. This was mediated by accumulation of the cell cycle mediators Cyclin D1 and c-Myc, resulting in increased replication stress–as measured by upregulation of CHK1, pH2AX–and apoptosis–assessed as BIM accumulation.

About ∼2% of childhood ALL cases are characterized by intrachromosomal amplification of chromosome 21 (iAMP21-ALL). Using integrated whole genome and transcriptome sequencing of 124 patients with iAMP21-ALL, including rare cases, Gao et al. (2023) identified subgroups of iAMP21-ALL based on the patterns of copy number alteration and structural variation. This large dataset enabled formal delineation of a 7.8 Mb common region of amplification harboring 71 genes, 43 of which were differentially expressed compared with non-iAMP21-ALL, including multiple genes implicated in the pathogenesis of acute leukemia such as DYRK1A, CHAF1B, ERG, HMGN1, and RUNX1. These results support a role for the overexpression of DYRK1A–and possibly other Hsa21 genes–in the pathogenesis of iAMP21-ALL.

Contrary to B-ALL, T-cell acute lymphoblastic leukemia (T-ALL) is extremely rare in people with Down Syndrome (Buitenkamp et al., 2014; Li et al., 2023; Maloney et al., 2010). The reasons underlying such reduced incidence remains a mystery. Nevertheless, DYRK1A’s ability to repress critical T-ALL leukemogenic pathways, such as NFAT (Catherinet et al., 2021) and NF-κB signaling (Wong et al., 2020; Grazioli et al., 2020) could help explain this phenotype.

Finally, DYRK1A could play a potential role in the regulation of other Hsa21-encoded genes implicated in DS-associated leukemias, like RUNX1 and RCAN1. RUNX1 is a transcription factor required for normal megakaryopoiesis and hematopoietic stem cell maintenance. Notably, germ-line mutations in RUNX1 have been linked to familial platelet disorders which may progress into myeloid cancers. RUNX1 somatic mutations and chromosomal rearrangements are frequently observed in myelodysplastic syndrome and leukemias of myeloid and lymphoid lineages (i.e., AML, ALL, and CML). Recently, Gialesaki et al. (2023) demonstrated that a shift in RUNX1 alternative splicing is key to DS-associated myeloid leukemia (DS-ML). In this context, they showed that expression of the RUNX1A isoform is elevated in patients with DS-ML, while mechanistic studies revealed that excessive RUNX1A synergizes with the GATA1s mutation during leukemogenesis by displacing the RUNX1C variant from its endogenous binding sites and inducing oncogenic programs. Wee et al. (2008) reported the *in vitro* phosphorylation of RUNX1 by DYRK1A. The authors speculate that such phosphorylation event is required for normal RUNX1 transcriptional function, as they might be disrupted in oncogenic variants, although a role in RUNX1 negative regulation (or degradation) was not assessed. As for the Regulator of Calcineurin 1 (RCAN1), it has been suggested to play a tumor suppressive role in leukemic cells and some solid tumor models [reviewed by Lao et al. (2022)]. Importantly, DYRK1A was shown to directly interact with and phosphorylate RCAN1 on Serine-112 and Threonine-192. Phosphorylation of RCAN1 Thr-192 enhanced its ability to inhibit the phosphatase activity of calcineurin, leading to reduced NFAT transcriptional activity (Jung et al., 2011). In a follow-up study, the authors suggested that phosphorylation of RCAN1 Thr-192 by DYRK1A induced its association into insoluble aggregates (Song et al., 2013). It remains unknown whether DYRK1A can regulate RCAN1 in blood or immune cells and if this mechanism might contribute to malignancy in leukemic cells.

**FIGURE 2:**
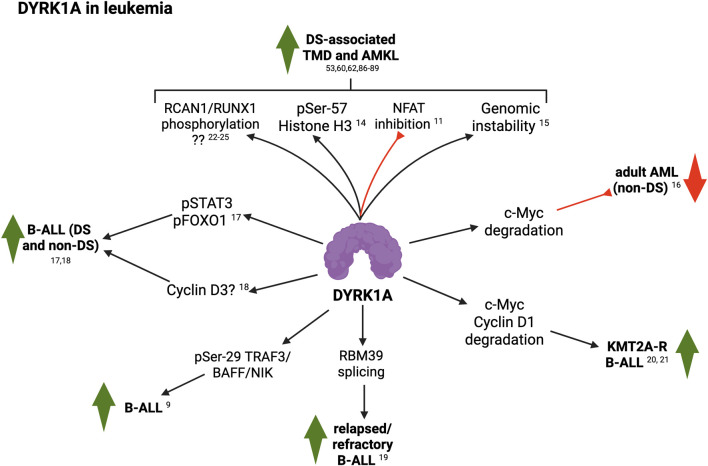
Summary of DYRK1A roles in leukemia (see text for details; created with BioRender). 9, Li et al. (2021); 11, Malinge et al. (2012); 14, Jang et al. (2014); 15, Chen et al. (2024); 16, Liu et al. (2014); 17, Bhansali et al. (2021); 18, Carey-Smith et al., (2022); 19, Jin et al. (2024); 20, Ayyadevara et al. (2025); 21, Ayyadevara et al. (2022); 22, Wee et al. (2008); 23, Lao et al. (2022); 24, Jung et al. (2011); 25, Song et al. (2013).

In conclusion, several lines of evidence support direct and complementary roles for DYRK1A in leukemia, both in people with and without T21 (Figure 2), and highlight its inhibition as a novel therapeutic opportunity against these malignancies. One interesting aspect that remains obscure is whether DYRK1A regulation of inflammation could also contribute to leukemic development and aggressiveness. As detailed in the previous section, DYRK1A fulfills a spectrum of pro-inflammatory functions. In recent years, many groups have shown that increased inflammatory signals are associated with poor survival outcomes in AML patients (Tsimberidou et al., 2008; Sanchez-Correa et al., 2013; Lasry et al., 2023; Rodriguez-Meira et al., 2023). Hence, how DYRK1A contributes to the hyperinflammatory state of people with DS, and how these dysregulated mechanisms reinforce DS-associated leukemias should be the focus of future efforts.

### DYRK1A in other inflammatory responses

Outside of the immune system, DYRK1A also appears to play diverse roles in inflammation. Upregulation of the Wnt pathway contributes to knee osteoarthritis (OA) by regulating osteoblast differentiation, increased catabolic enzymes, and expression of inflammatory mediators. Deshmukh et al. (2019) observed that the Wnt inhibitor, lorecivivint (SM04690) actually blocked the kinases DYRK1A and CLK2 (CDC-like kinase 2) and showed that DYRK1A knockdown was sufficient for anti-inflammatory effects, while combined DYRK1A/CLK2 downregulation enhanced this effect. Using the monosodium iodoacetate (MIA)-induced rat OA model, lorecivivint inhibited production of inflammatory cytokines and cartilage degradative enzymes, resulting in increased joint cartilage, decreased pain, and improved weight-bearing function. In follow-up studies, this group conducted Phase 2 and 3 randomized clinical trials of lorecivivint (Yazici et al., 2020; Yazici et al., 2021) (NCT02536833; NCT03122860; NCT04520607) and demonstrated the efficacy of lorecivivint on patient-reported outcomes in subjects with knee OA. More recently, these findings have been challenged by Liu et al. (2023), who used an OA mouse model by destabilized medial meniscus (DMM) surgery in wild-type and chondrocyte-specific DYRK1A knockout (DYRK1A-cKO) animals, and suggested that DYRK1A expression can delay–not promote–disease progression in this murine model, likely through its ability to stabilize EGFR/ERK signaling. Lastly, increased expression of DYRK1A has also been reported both in the synovial tissues of rheumatoid arthritis (RA) patients and in a TNF-α-induced fibroblast-like synoviocyte (FLS) activation model (Guo et al., 2018). In this context, DYRK1A knockdown inhibited TNF-α-induced FLSs proliferation and migration/invasion ability. Given that DYRK1A has also been shown to play important roles in bone homeostasis (Otte and Roper, 2024), future studies should elucidate the exact implications of DYRK1A in inflammation versus other regulatory processes in these conditions.

Importantly, around 50% of children with DS manifest hearing loss due to otitis media with effusion (OME). Using a panel of mouse models harboring duplications of small regions of Mmu16 syntenic to Hsa21, Tateossian et al. (2025) identified *Dyrk1a* as a key gene underlying this condition and demonstrated that normalization of *Dyrk1a* gene dosage restored the wild-type phenotype. Mechanistically, the authors observed that *Dyrk1a* triplication leads to middle ear inflammation and vascular leak through a cross-talk with TGF-β signaling and its impact on proinflammatory cytokines IL-6 and IL-17, as well as raised VEGF levels in the middle ear accompanied by increased expression of the Hypoxia-inducible factor 1-alpha (Hif1a) transcription factor.

Lan et al. (2024) reported a role for DYRK1A in hypoxia-induced pulmonary artery remodeling and hypertension. through a mechanism invovlving the STAT3/Pim-1/NFAT pathway. Additionally, Suginobe et al. (2024) observed upregulation of DYRK1A signaling in samples of DS-associated pulmonary arterial hypertension (Suginobe et al., 2024). Finally, using a mouse model of diabetes and human keratinocyte cell lines, Hu et al. (2024) observed that miR-221-3p directly targets DYRK1A and blocks glucose-induced DYRK1A-mediated STAT3 signaling and skin inflammation.

In the mouse brain, overexpression of Dyrk1A (TgDyrk1A mouse model) was found to stabilize IκBα protein levels and increase cytoplasmic sequestration of NFκB p65/RelA, while Dyrk1A-deficient mice exhibited the reverse outcomes (Latour et al., 2019), suggesting a role for DYRK1A as a negative regulator of the NFκB pathway. In parallel, Ju et al. (2022) investigated the role of Dyrk1A in neuroinflammation using the murine BV2 microglia cell line induced with lipopolysaccharide (LPS). Contrary to Latour et al. (2019), this group showed significant induction of Dyrk1A expression by LPS, while Dyrk1A inhibition by harmine or siRNA silencing significantly reduced the production of proinflammatory factors such as reactive oxygen species (ROS) and tumor necrosis factor-α (TNF-α), among others. *In vivo*, harmine treatment decreased the expression of inflammatory proteins in the cortex and hippocampus in mice injected with LPS into the cerebral ventricle. At the molecular level, they found that Dyrk1A suppression effects are likely due to inhibition of the TLR4/NFκB signaling pathway in LPS-induced neuroinflammation (Ju et al., 2022). Similarly, He et al. (2024) found that miR-192-5p downregulates Dyrk1A levels, which was necessary to attenuate neuroinflammation and neural apoptosis in the ischemic stroke murine model of middle cerebral artery occlusion (MCAO). In summary, DYRK1A overexpression in T21 has been associated with many neurodevelopmental, behavioral and neurodegenerative alterations, including DS-related Alzheimer’s disease (DS-AD). Notably, neuroinflammation has recently emerged as a prominent factor in the manifestation of AD. Nevertheless, a potential direct role for DYRK1A in AD-related neuroinflammation has not been examined yet.

**FIGURE 3:**
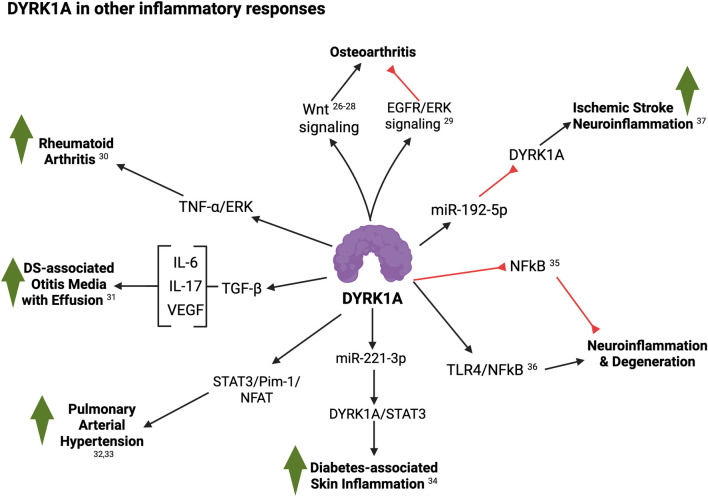
Summary of DYRK1A roles in other inflammatory responses (see text for details; created with BioRender). 26, Deshmukh et al. (2019); 27, Yazici et al., 2020; 28, Yazici et al., 2020; 29, Liu et al. (2023); 30, Guo et al. (2018); 31, Tateossian et al. (2025); 32, Lan et al. (2024); 33, Suginobe et al. (2024); 34, Hu et al. (2024); 35, Latour et al. (2019); 36, Ju et al. (2022); 37, He et al. (2024).

Altogether, these reports suggest a widespread and conserved role for DYRK1A as a critical mediator of inflammation (Figure 3). Lastly, DYRK1A has been shown to play important roles in endothelial homeostasis and vascular function (Suginobe et al., 2024; Rozen et al., 2018; Cho et al., 2019), having critical implications in inflammatory responses. In parallel, DS has been deemed as an Interferonopathy (Galbraith et al., 2023; Chung et al., 2021), where IFN signaling is exacerbated and contributes to many pathophysiological characteristics of T21. While IFN-dependent responses have received much attention, very little is known about whether and how other inflammatory responses might synergize with it and predispose individuals towards certain disorders and phenotypes. The reports summarized in this article strongly support an IFN-independent role for DYRK1A in inflammation and autoimmunity, which may drive or further potentiate a dysregulated immune microenvironment. Future efforts should delineate the feasibility and relevance of pharmacologically targeting DYRK1A signaling as a therapeutic strategy for correcting these inflammatory disorders in individuals with Down syndrome.

### DYRK1A in viral infections

Despite the abundant reports demonstrating that DYRK1A plays many different functions during viral infections, a synthesis of such activities, and their implications in DS health and disease, is lacking. In this section, we thus summarize the evidence supporting the pleiotropic and context-dependent roles of this kinase in viral biology, and its potential links to T21 infection susceptibility. For years, DYRK1A has been known to bind, phosphorylate and/or regulate different viral proteins implicated in viral entry, replication, transcription and pathogenesis, including human immunodeficiency virus HIV-1 and severe acute respiratory syndrome coronavirus 2 (SARS-CoV-2). DYRK1A appears to exert pleiotropic–and even opposing–functions in a virus type-specific manner. Regarding HIV-1, Bol et al. (2011) reported a strong correlation between *in vitro* viral replication and the *DYRK1A* single nucleotide polymorphism (SNP) rs12483205 in monocyte-derived macrophages, and found this SNP to be associated with HIV-1 disease progression *in vivo* in two independent cohort studies, suggesting that DYRK1A may in fact be involved in HIV-1 replication in macrophages. Subsequently, Booiman et al. (2015) demonstrated that DYRK1A controls HIV-1 provirus transcription by repressing NFAT signaling. Downregulation or inhibition of DYRK1A increased LTR-driven transcription and viral replication in cell lines and primary PBMCs. Furthermore, DYRK1A blockade resulted in efficient reactivation of latent HIV-1 provirus. Mechanistically, inhibition of DYRK1A resulted in nuclear accumulation of NFAT and increased NFAT binding to the viral LTR, thus enhancing viral transcription. In previous work, Kyei et al. (2015) showed that cyclin L2 is required for HIV replication in macrophages. In a follow-up study, this group (Kisaka et al., 2020) demonstrated that DYRK1A phosphorylates cyclin L2, while mutation of its DYRK1A-target sites or depletion of DYRK1A significantly stabilized cyclin L2 and increased HIV-1 replication in macrophages (Kisaka et al., 2020). Depletion of cyclin L2 decreased HIV-1 replication, supporting the idea that DYRK1A controls cyclin L2 expression, leading to restriction of HIV replication.

Earlier, increased expression of DYRK1A had been observed in keratinocytes immortalized with the oncogenic human papillomavirus HPV16 and in cervical cancer samples as compared to uninfected counterparts (Chang et al., 2007). Their results suggested that higher DYRK1A expression promoted by this oncovirus may prevent cancer cell apoptosis through regulation of the FOXO1 transcription factor. Additionally, DYRK1A interacts with and phosphorylates HPV16’s oncoprotein E7, both *in vitro* and *in vivo* (Liang et al., 2008). This interaction greatly increased E7 levels. Moreover, Yang et al. (2015) found that expression of the DYRK1A-targeting miR-1246 was negatively correlated to HPV16 infection and cervical cancer clinical stage. Interestingly, they observed that knock-down of HPV16 E6 protein resulted in upregulation of miR-1246 and concomitant reduction of DYRK1A, while the opposite effect was seen upon E6 protein overexpression.

The adenovirus oncoprotein E1A (early region 1A) induces cell proliferation, oncogenic transformation and viral replication through interaction with multiple transcriptional regulatory complexes and signaling proteins. Komorek et al. (2010) first showed that DYRK1A can interact with E1A. Later, Cohen et al. (2013) and Glenewinkel et al. (2016) suggested that interaction of E1A with DYRK1A–mediated by its binding partner DCAF7 (Glenewinkel et al., 2016)– might antagonize Ras-mediated transformation. Hutterer et al. (2017) reported a block of viral replication at the early-late stage of human cytomegalovirus (HCMV) gene expression by several DYRK inhibitors. In addition, they confirmed that other types of viruses, such as rhesus macaque cytomegalovirus (RhCMV), varicella-zoster virus (VZV) and herpes simplex virus (HSV-1), also exhibited strong sensitivity to DYRK blockade. In a follow-up study, Hamilton et al. (2018) observed that HCMV-infected placental cells showed upregulation, accumulation and re-localization of DYRK1A and DYRK1B proteins to areas of cytoplasmic virion assembly complexes and nuclear viral replication compartments, respectively, while DYRK inhibitors significantly inhibited HCMV replication. More recently, Egilmezer et al. (2024) confirmed the relocalization of DYRK1A into cytoplasmic virion assembly complexes of HCMV-infected primary human astrocytes and placental cells. Finally, Wang et al. (2023) reported that siRNA-mediated downregulation or pharmacologic inhibition of DYRK1A blocked macropinocytosis-dependent entry of pseudorabies virus (PRV).

Regarding coronaviruses (CoVs), Wei et al. (2021) conducted genome-wide CRISPR screens to identify therapeutic targets for SARS-CoV-2 and related CoVs, and identified DYRK1A among the genes that potentially mediate entry of SARS-lineage viruses. Grodzki et al. (2022) independently confirmed these results, and further showed that the DYRK1A inhibitor harmine suppressed *in vitro* SARS-CoV-2 infection. In a follow-up study from the former group, Strine et al. (2023) demonstrated that DYRK1A regulates transcription of the angiotensin-converting enzyme 2 (ACE2) receptor and of the dipeptidyl peptidase-4 (DPP4). SARS-CoVs use ACE2 as a receptor for infection, whereas Middle East Respiratory Syndrome CoVs (MERS-CoVs) bind to the dipeptidyl peptidase-4 (DPP4) receptor (Li et al., 2003; Letko et al., 2020; Hoffmann et al., 2020; Raj et al., 2013; Qing et al., 2020). Mechanistically, they found that DYRK1A induced *ACE2* and *DPP4* transcription and expression by altering chromatin accessibility at their promoters and enhancers in a kinase-independent manner. More recently, a genome-wide CRISPR knockout screen by Mao et al. (2024) reported that the host factors TRAF3, DYRK1A, and RAD54L2 form a so-called ‘TDR’ complex to regulate the expression of ACE2, while knocking out any of these factors reduced *ACE2* mRNA levels and inhibited the cellular entry of SARS-CoV-2. In support of this, Fu et al. (2024) observed that DYRK1A–but not its kinase activity–is also required for viral entry and replication of transmissible gastroenteritis CoVs (TGE-CoVs). Additionally, these authors validated the proviral roles of DYRK1A in mouse hepatitis virus, porcine deltacoronavirus, and porcine sapelovirus, demonstrating that DYRK1A is an essential host factor for infections by multiple viruses.

At last, through a combination of genetic and chemical approaches using hepatitis B virus (HBV)-infected hepatocytes, Pastor et al. (2024) found that DYRK1A positively regulates the production of HBV RNAs. DYRK1A bound to the HBV episomal genome and stimulated the production of HBV nascent RNAs. Finally, they showed that DYRK1A upregulates the HBV enhancer 1/X promoter activity in a sequence-dependent manner, proposing that DYRK1A is a proviral factor that may participate in the life cycle of HBVs.

**FIGURE 4:**
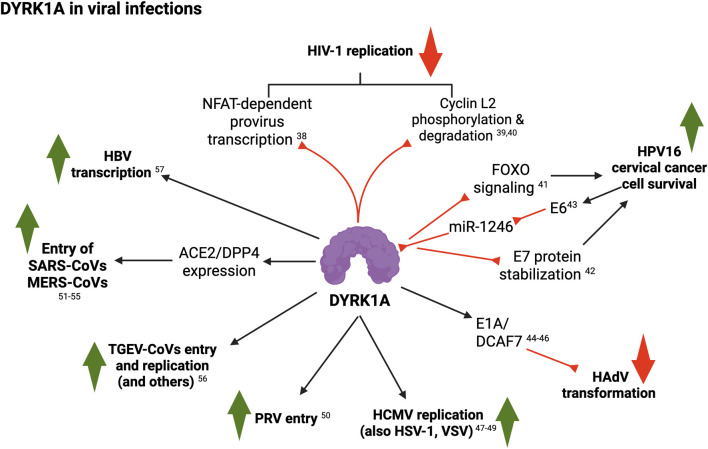
Summary of DYRK1A roles in viral infections (see text for details; created with BioRender). 38, Booiman et al. (2015); 39, Kyei et al. (2015); 40, Kisaka et al. (2020); 41, Chang et al. (2007); 42, Liang et al. (2008); 43, Yang et al. (2015); 44, Komorek et al. (2010); 45, Cohen et al. (2013); 46, Glenewinkel et al. (2016); 47, Hutterer et al. (2017); 48, Hamilton et al. (2018); 49, Egilmezer et al. (2024); 50, Wang et al. (2023); 51, Wei et al. (2021); 52, Grodzki et al. (2022); 53, Strine et al. (2023); 54, Qing et al., 2020; 55, Mao et al. (2024); 56, Fu et al. (2024); 57, Pastor et al. (2024); 58, Fu et al. (2024).

In summary, several lines of work report pleiotropic–and potentially synergistic–roles for DYRK1A in the regulation of viral entry, replication and pathogenesis (Figure 4).

Several epidemiological studies indicate that individuals with DS present lower incidences of viral disease, but more severe responses when present (Malle et al., 2022; Gansa et al., 2024; Fitzpatrick et al., 2022). People with DS display 5-10x higher risk of hospitalization and death by SARS-CoV-2 (Clift et al., 2021; De Toma and Dierssen, 2021; Espinosa, 2020; Malle et al., 2021; Illouz et al., 2021; Hüls et al., 2021), while pneumonias remain a leading cause of mortality in adults with DS (Chenbhanich et al., 2019; Santoro et al., 2021). Notably, people with T21 exhibit lower prevalence of other respiratory infections (including influenza) and sexually transmitted infections (including genital herpes, HIV/AIDS and human papillomavirus), among many other infectious diseases (Gansa et al., 2024; Fitzpatrick et al., 2022). The mechanisms underlying such reduced susceptibility to certain viral infections are varied and far from full elucidation–with heightened IFN responses suggested to play a main role. However it is interesting to note that in some of these infections with lower incidence in people with DS population, DYRK1A plays antiviral functions (e.g., HIV-1 and HPV), whereas in those that are more frequent or severe in this group (such as SARS-CoV-2), DYRK1A exerts proviral activities. Hence, overexpression of DYRK1A could be at the root of the heterogeneous predisposition to viral infections and pathogenesis found in the T21 population. In conclusion, DYRK1A is emerging as multifunctional critical modulator of viral infection, and therefore an attractive therapeutic target for the management of specific viral infections in people with Down syndrome and beyond.

## Conclusion and future prospects

DYRK1A is a master regulatory kinase at the crossroads of many different signaling cascades. Its implications in health and disease have been the focus of long-term research efforts and are poised to transform our knowledge and the therapeutic options for a wide spectrum of disease states. This is further emphasized in the context of T21, where DYRK1A is known or suspected to play critical roles in many developmental and physiological alterations frequently associated with DS. This is the first synthesis of the literature summarizing the roles of this protein in blood and immune function, as well as in leukemia, inflammatory responses and viral infections. Our review highlights a few controversies, where different groups have suggested seemingly contradictory results. While these could be partly explained by different experimental conditions and models, the conclusion is that more research is needed to fully elucidate the roles of DYRK1A in these and other paradigms. As a more general message, we can outline that DYRK1A is indeed a critical mediator of diverse processes in blood cell development and function, and its dysregulation certainly is a driver of pathologic states such as leukemia, autoinflammatory disorders and viral susceptibility, all of which are extremely relevant for individuals with Down syndrome. Hence, emerging DYRK1A inhibitory molecules might represent innovative therapeutic strategies for the management of immune-related conditions frequently associated with T21. In this regard, we kindly refer the reader to several excellent reviews on the progress and characterization of novel DYRK1A inhibitory biomolecules (Yang et al., 2023; Murphy et al., 2024; Deau et al., 2023; Liu T. et al., 2022). Our review should provide useful guidance for the research community and will hopefully inspire new projects aiming at better understanding the implications of DYRK1A in hematologic homeostasis, inflammation and infection.
